# Entropy-Based Analysis of Acoustic Emission Evolution During Tensile and V-Bending Deformation of AZ31B Magnesium Alloy Sheets

**DOI:** 10.3390/ma19183964

**Published:** 2026-09-18

**Authors:** In-Gyu Choi, Jung-Sik Yoon, Chang-Whan Lee

**Affiliations:** 1 Department of Mechanical Information Engineering, Seoul National University of Science and Technology, Seoul 01811, Republic of Korea; igchoi@seoultech.ac.kr (I.-G.C.); mae04064@seoultech.ac.kr (J.-S.Y.); 2 Department of Mechanical Engineering, Seoul National University of Science and Technology, Seoul 01811, Republic of Korea

**Keywords:** acoustic emission, magnesium alloy, Shannon entropy, continuous wavelet transform, deformation-stage segmentation, unsupervised clustering

## Abstract

This study presents an entropy-based framework for segmenting and characterizing the deformation behavior of AZ31B magnesium alloy sheets using acoustic emission (AE) signals obtained during tensile and V-bending tests. Shannon entropy was calculated from four AE parameters (duration, count, signal energy, and frequency centroid). The entropy values were normalized and averaged to identify transitions between deformation stages. Individual AE waveforms were transformed into normalized time–frequency images using the continuous wavelet transform (CWT), and quantitative features extracted from these images were used for Random Forest classification of the entropy-defined stages. Internal five-fold cross-validation yielded accuracies of 94.6% and 96.42% for tensile and V-bending deformation, respectively, indicating that the stages were distinguishable in the CWT-based feature space. Hierarchical density-based spatial clustering of applications with noise (HDBSCAN) separated fracture-associated AE signals during tensile deformation. The results indicate that the proposed method can characterize deformation-stage evolution and provide additional information on fracture-associated AE responses in AZ31B sheets.

## 1. Introduction

Acoustic emission (AE) is a non-destructive testing (NDT) technique that detects transient elastic waves generated by micro-deformation and damage processes within materials, and has been widely used for real-time monitoring of structural integrity. When localized deformation, crack initiation, crack propagation, frictional sliding, or fracture occurs in a material, part of the released strain energy propagates as elastic waves and can be detected by piezoelectric sensors attached to the specimen or structure [1]. Thus, AE monitoring enables real-time and in situ detection of damage evolution under loading or operating conditions [2]. In particular, AE parameters such as counts, amplitude, energy, duration, rise time, and frequency components provide useful information for characterizing the initiation and progression of internal damage mechanisms [3].

AE has been widely applied to structural health monitoring and damage diagnosis because it enables real-time detection of deformation and fracture-associated events [4]. Roberts et al. [5] demonstrated a strong correlation between AE signal characteristics and fatigue crack propagation. AE has also been applied to fracture characterization [6], damage evaluation in industrial facilities [7], and crack initiation analysis under bending conditions [8]. These studies established AE as an effective diagnostic tool for monitoring damage evolution in engineering materials. Strantza et al. [9] further demonstrated the applicability of AE parameters to fatigue crack propagation in additively manufactured metallic components.

Beyond structural applications, AE has also been applied to metal forming processes, where plastic deformation, twinning, and microcrack initiation generate characteristic AE responses. Behrens et al. [10,11,12] investigated AE behavior during cold forging and sheet forming and reported deformation-stage-dependent signal characteristics. Mukhopadhyay et al. [13] studied AE monitoring during hot forging, while Zhao et al. [14] combined AE with digital image correlation to analyze damage evolution during sheet blanking. Pomponi et al. [15] further demonstrated the use of clustering techniques for real-time classification of AE signals.

As AE datasets have become increasingly complex and high-dimensional, machine learning (ML) techniques have been actively introduced to extract meaningful information from such signals. Wisner et al. [16] applied Gaussian mixture models (GMM) to cluster AE features associated with fracture behavior. Muir et al. [17] demonstrated the capability of data-driven approaches to link AE waveform characteristics with underlying damage mechanisms. In the context of metal forming, Kwon et al. [18] utilized wavelet-based energy mapping and unsupervised learning for ductile fracture detection, whereas Barile et al. [19] applied continuous wavelet transform (CWT) and convolutional neural networks (CNNs) to classify deformation behavior in additively manufactured alloys. Yu et al. [20] showed that clustering of AE features could distinguish signal characteristics associated with yielding, work hardening, and fracture. These studies demonstrate the potential of data-driven methods for AE signal characterization, but they mainly focus on separating predefined signal groups or damage states rather than tracking the continuous evolution of AE behavior during deformation.

AE parameters exhibit different distributions as deformation and damage evolve, and these changes cannot always be represented adequately by individual AE parameters. Shannon entropy provides a quantitative measure of the uncertainty and complexity of such parameter distributions [21,22]. Chai et al. [23] demonstrated that acoustic emission entropy can be used as an effective quantitative parameter for identifying crack initiation and assessing fatigue crack growth in 316LN stainless steel. Karimian and Modarres [24] used waveform analysis and information entropy-based features to cluster AE signals in CFRP laminates, successfully distinguishing matrix-dominated and fiber-dominated damage signals. Barile et al. [25] showed that entropy-based AE waveform analysis, combined with spectral density and wavelet packet transform, can be used to identify damage processes in CFRP composites. Ahmed and Dutta [26] used Shannon entropy-based AE entropy to identify successive damage stages during fiber pull-out in ultra-high performance concrete, demonstrating that entropy-based AE parameters can effectively characterize damage evolution and critical damage transitions. Unlike individual AE parameters, which describe specific aspects of AE activity such as amplitude, energy, or duration, entropy provides a quantitative measure of changes in the overall distribution and complexity of multiple AE parameters. Therefore, the entropy-based approach enables the characterization of collective AE evolution during deformation rather than relying on individual signal features alone.

In this study, AZ31B was selected because its deformation behavior has been well established, making it suitable for evaluating changes in AE responses during plastic deformation, localization, and fracture. However, the evolution of AE signals during these stages has not been sufficiently quantified. In particular, few studies have compared entropy-based AE responses under tensile and bending deformation.

To address this gap, this study investigates how the collective AE response evolves as deformation progresses from plastic deformation to localization and fracture in AZ31B magnesium alloy sheets under tensile and V-bending conditions. Rather than attempting to directly identify individual microscopic deformation mechanisms from AE signals, Shannon entropy was calculated from multiple AE parameters to quantify time-dependent changes in the distribution and complexity of AE activity. The resulting entropy evolution was then used to identify transitions between macroscopic deformation stages.

To further characterize the entropy-defined deformation stages, CWT-based features [27] were extracted from individual AE signals. Random Forest classification was used to examine whether the entropy-defined stages exhibited distinguishable characteristics in the CWT-based feature space, while hierarchical density-based spatial clustering of applications with noise (HDBSCAN) was applied to explore AE events occurring near final fracture. These methods were used as complementary tools to characterize the AE response associated with the entropy-defined stages rather than to identify specific deformation mechanisms. By applying the same entropy-based framework to both tensile and V-bending deformation, this study investigates how AE signals evolve under different deformation modes.

## 2. Experimental Methods

### 2.1. Tensile and Bending Tests

**Figure 1:**
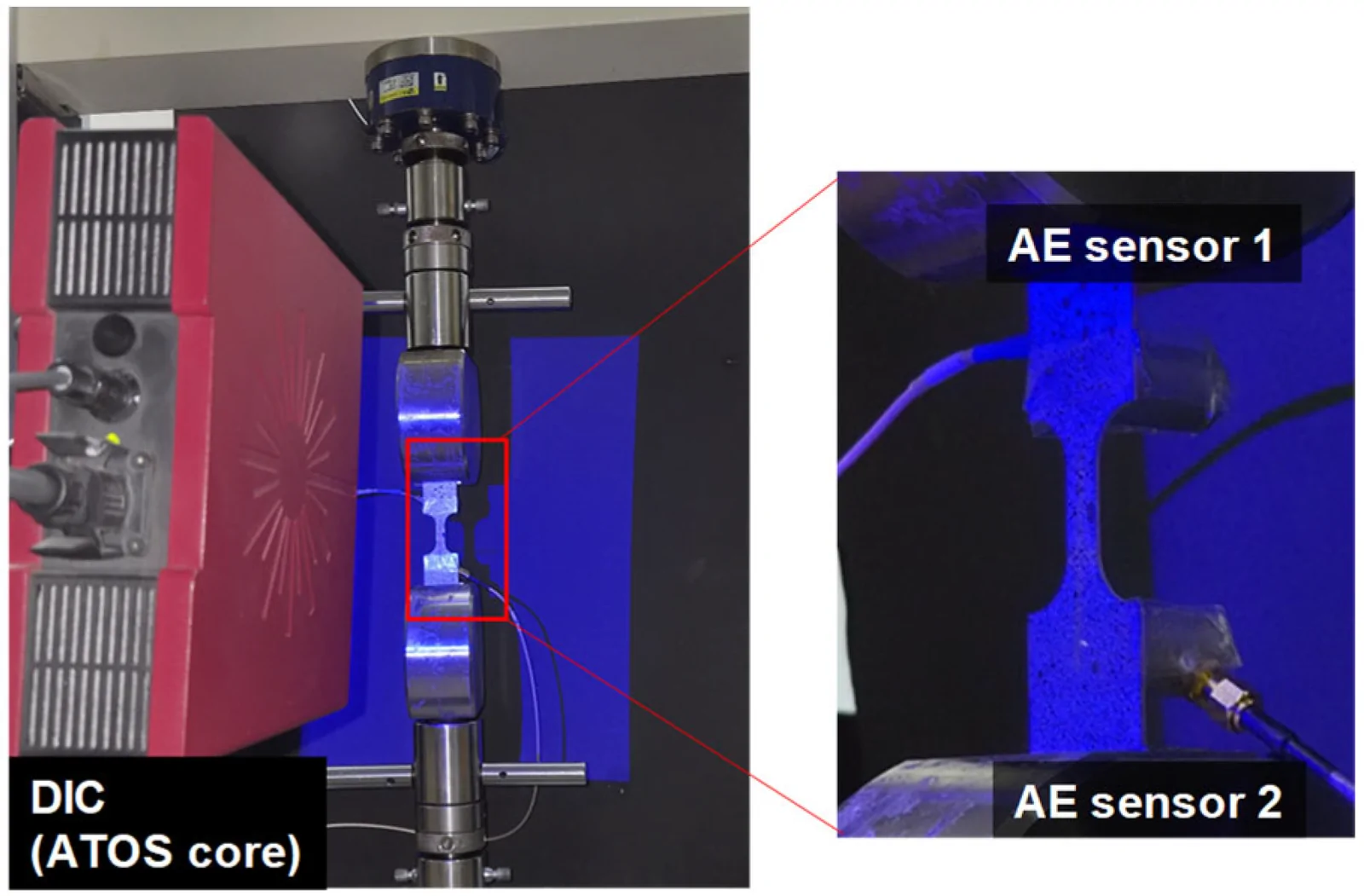
Experimental setup for the tensile test of magnesium alloy sheets.

**Figure 2:**
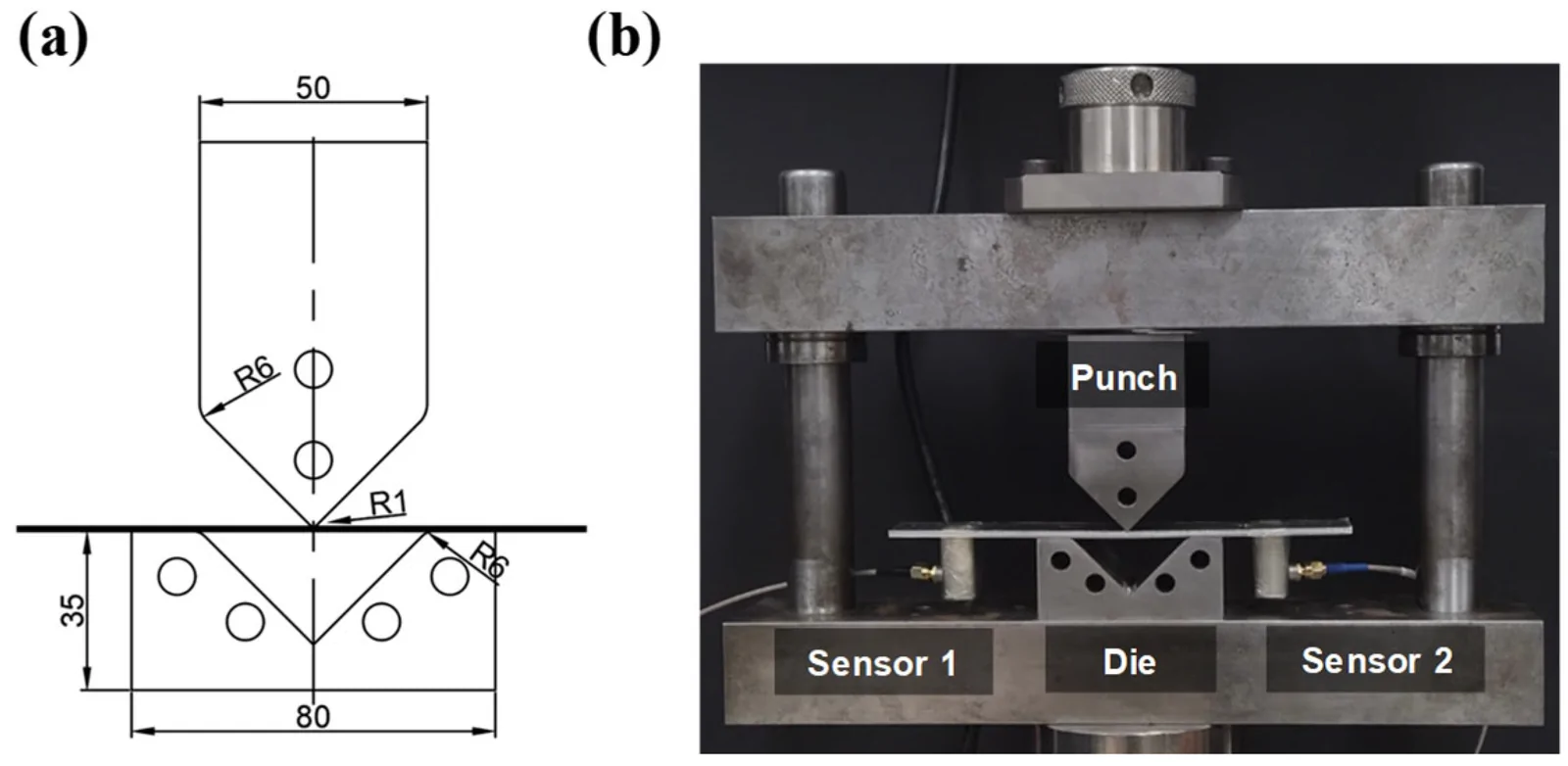
Experimental setup for the V-bending test of magnesium alloy sheets (dimensions are in mm). The schematic drawing (**a**) and experimental setup (**b**).

Tensile tests were performed using a universal testing machine (MINOS-020, MTDI, Daejeon, South Korea) at a constant crosshead speed of 5 mm/min, as shown in Figure 1. Sheet specimens with thicknesses of 1 mm and 3 mm were prepared, each having a gauge length of 25 mm and a width of 12.5 mm. V-bending tests were performed under displacement control at a constant punch speed of 5 mm/min. The schematic drawing and experimental setup for the V-bending test are shown in Figure 2. Each specimen (200 mm × 30 mm, thicknesses of 1 mm and 3 mm) was placed on a V-die with an opening angle of 90° and a corner radius of 6 mm. A punch with a tip radius of 1 mm was used to apply the forming load. All specimens were prepared with their longitudinal direction parallel to the rolling direction of the sheet.

For the tensile tests, two AE sensors (IDK-AES-R150, IDK, Daejeon, South Korea) with a resonant frequency of 150 kHz were mounted on the upper and lower shoulder regions of the specimen. For the V-bending tests, the sensors were attached to the flat regions on the back side of the specimen. Vacuum grease was applied at the interfaces between the sensor and material to ensure stable acoustic coupling. Sensors were secured using adhesive tape. In the V-bending tests, to reduce friction, grease was applied to the die shoulder and the punch. This lubrication minimized AE signals associated with friction [28].

AE signals were acquired using a digital data acquisition (DAQ) system connected to the AE sensors. The acquisition settings are summarized in Table 1. Sensor responses were verified using pencil lead break tests. Two AE sensors were used to verify the consistency of signal detection and acoustic coupling at different positions on the specimen rather than for source localization. As the two sensors produced similar responses, only the data from Sensor 1 were used for further analysis. Each test condition was repeated three times, and the repeated tests showed similar load histories, fracture times, and overall AE trends. One representative dataset from each condition was therefore selected for the detailed entropy analysis.

To reduce the influence of measurement noise, a threshold of 40 dB and a band-pass filter of 20–1000 kHz [20] were applied during AE acquisition to suppress low-level background noise and out-of-band signal components. The hit lockout time (HLT) was set to 4000 μs to prevent residual oscillations following an AE event from being repeatedly detected as separate hits. The maximum hit duration (MHD) was set to 5000 μs. Therefore, AE events exceeding this duration were recorded at the predefined upper limit of 5000 μs.

### 2.2. Materials

**Figure 3:**
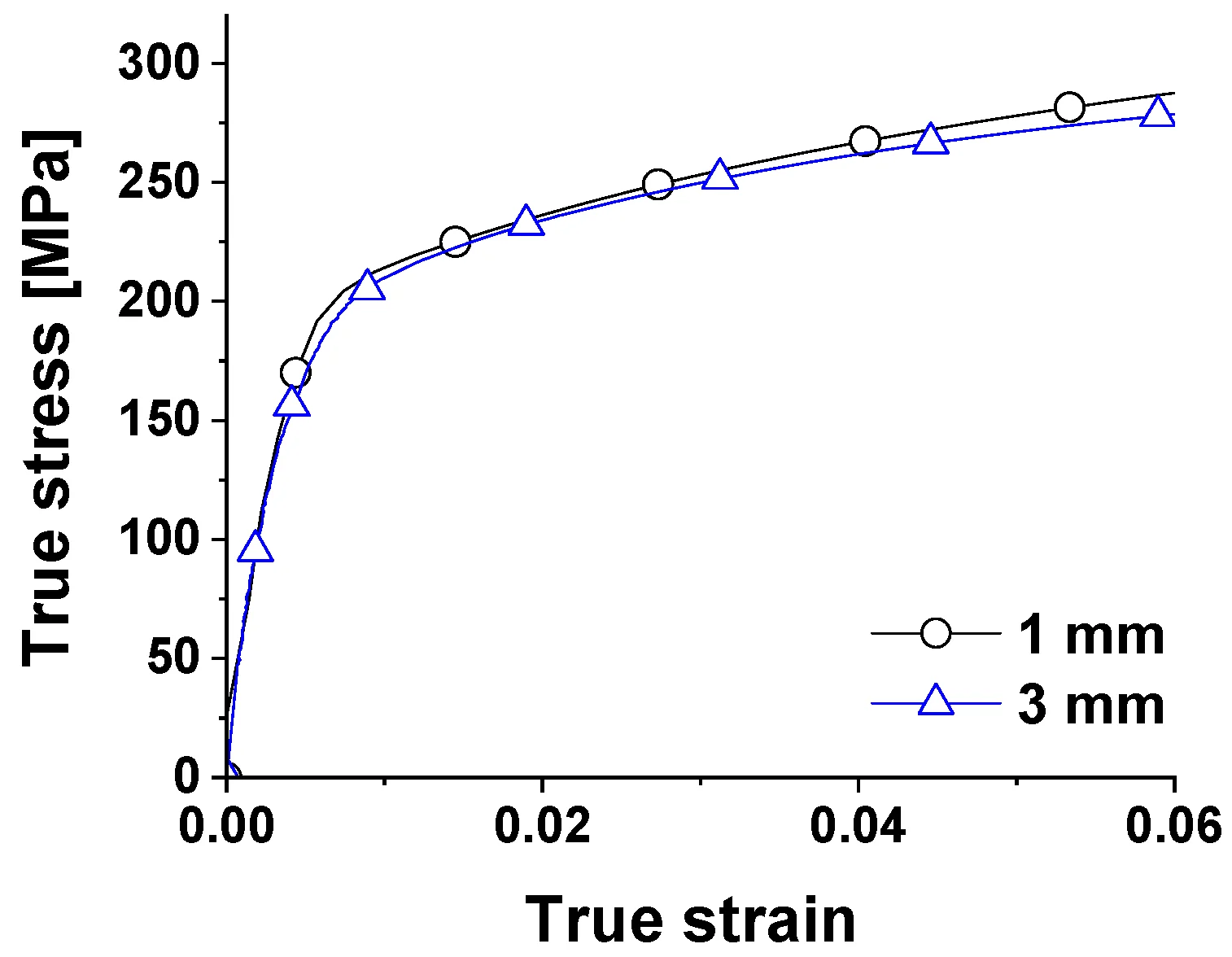
True stress–strain curves of magnesium alloy sheets with thicknesses of 1 mm and 3 mm.

In this study, AZ31B magnesium alloy sheets with thicknesses of 1 mm and 3 mm were used. The corresponding stress–strain curves obtained from the tensile tests are presented in Figure 3. The density of the material was 1850 kg/m^3^ and the Poisson’s ratio was 0.3. Tensile testing revealed elastic moduli of 43.4 GPa and 46.1 GPa for the 1 mm- and 3 mm thick sheets, respectively. The corresponding yield strengths were 191.7 MPa and 178.75 MPa.

### 2.3. Shannon Entropy

Traditional AE analyses often rely on individual parameters such as amplitude, duration, or energy to characterize material behavior. However, single-parameter-based approaches are frequently insufficient for reliable discrimination of deformation stages, as each parameter exhibits different sensitivities to local deformation conditions and measurement noise.

In this study, four AE parameters–duration, count, signal energy, and frequency centroid–were used to represent the time- and frequency-domain characteristics of the signals. These parameters describe different aspects of AE activity and vary with the deformation stage. Shannon entropy was calculated for each parameter to quantify changes in signal complexity and uncertainty, as defined in Equation (1):

(1) $$H=-\sum_{i=1}^{n}p_{i}log(p_{i})$$

where $p_{i}$ denotes the probability distribution of parameter values within a given time window. The entropy data were normalized to remove differences in scale and then averaged to obtain a normalized mean entropy curve. Missing values were replaced with zeros before smoothing. A sliding window of 0.2 s was used for the tensile test, whereas a 2 s window was used for the V-bending test because of its longer deformation duration and larger number of AE events. The window lengths and moving-average parameters were selected considering the different time scales of the tensile and V-bending tests, with the aim of reducing short-term fluctuations while preserving overall entropy evolution. The normalized mean entropy curve was then smoothed using a moving-average filter with window sizes of 5 samples for the tensile test and 20 samples for the V-bending test. Inflection points in the normalized mean entropy curve were subsequently identified and used as boundaries for defining deformation stages in both tensile and bending tests.

### 2.4. CWT Image Generation and Feature Extraction

**Figure 4:**
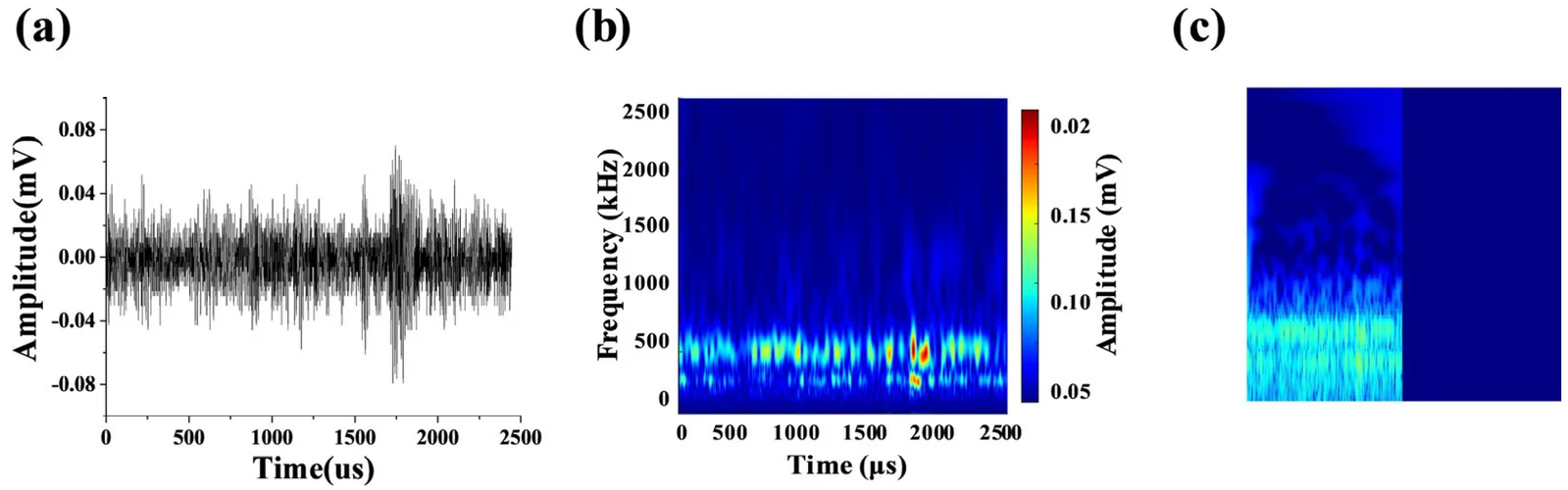
Comparison of a single AE signal and related CWT images: (**a**) Time-amplitude relation, (**b**) CWT image and (**c**) normalized CWT image used for feature extraction. The blue region on the right side of (**c**) represents the zero-padded interval corresponding to the absence of AE waveform data.

Figure 4 shows a representative AE hit recorded during the tensile test as a time–amplitude waveform, which describes the variation in signal amplitude over time. Although such time-domain AE signals can be analyzed in the frequency domain using the Fourier transform, this approach does not simultaneously provide information on the temporal variation in frequency components. Therefore, the continuous wavelet transform (CWT) was applied in this study to convert the non-stationary AE waveforms into time–frequency representations.

The CWT was performed using the default analytic Morse wavelet in MATLAB R2024a (The MathWorks, Inc., Natick, MA, USA). Each AE waveform was transformed into a two-dimensional time–frequency–magnitude image. In the generation of the CWT images, a fixed time span of 5000 μs, corresponding to the maximum hit duration used during AE acquisition, and a frequency range up to 600 kHz were used to generate CWT images of consistent size. For AE signals with durations shorter than 5000 μs, the remaining portion of the time span was zero-padded to maintain a consistent image size. The amplitude ranges were set to 10^−4^–10 mV for tensile deformation and 10^−3^–10^1.4^ mV for bending deformation. In addition, the signal magnitude was represented on a logarithmic scale to improve the visualization of both low- and high-intensity signal components. Accordingly, the blue region on the right side of the normalized CWT image in Figure 4c corresponds to the zero-padded region where no AE waveform data were recorded. The normalized CWT image was used as an image-based representation for subsequent feature extraction; therefore, the original time and frequency coordinates are not explicitly displayed in Figure 4c.

### 2.5. Method for Random Forest Classification of Deformation Stages

AE signals are inherently non-stationary and sensitive to noise; therefore, classification based on a single AE parameter or an individual event may be unstable. To address this issue, multiple features were extracted from the CWT images obtained from each AE segment and integrated into feature vectors suitable for machine-learning-based classification. These features were used to construct numerical representations of the CWT images for subsequent classification.

In this study, a total of 17 features were extracted from each normalized CWT image to characterize AE signal characteristics in the time–frequency domain. As summarized in Table 2, these features include statistical descriptors, GLCM-based texture features, histogram-based percentile features, and frequency-domain spectral features. These feature groups were selected to describe complementary characteristics of the CWT images, including signal intensity, spatial distribution, intensity distribution, and spectral characteristics. They were used as descriptive features of the CWT images rather than as direct indicators of specific AE source or deformation mechanisms.

Since features extracted from a single CWT image may be affected by local waveform variation, a sub-window strategy was employed. Twenty consecutive CWT images were grouped into one sub-window, and the mean, standard deviation, and maximum of each of the 17 features were calculated. Consequently, a 51-dimensional feature vector (17 × 3) was obtained for each sub-window and used as input to the Random Forest classifier. The entropy-defined deformation stages were used as supervised labels.

Random Forest was selected because it can effectively handle nonlinear relationships among multiple heterogeneous features and is suitable for classification with relatively limited datasets. In addition, it requires fewer assumptions regarding the distribution and scaling of input features and is less prone to overfitting than a single decision tree owing to its ensemble structure. In the present study, Random Forest was used primarily to examine whether the entropy-defined deformation stages were distinguishable in the CWT-based feature space, rather than to develop a generalized predictive model.

The deformation-stage labels determined from the entropy-based segmentation were assigned to the corresponding sub-window feature vectors and used as supervised labels for classifier training. Because the number of feature vectors differed among deformation stages, class imbalance was observed in the training dataset. In particular, the number of samples in the later stage near fracture was relatively limited because AE activity changed abruptly within a short time interval.

To reduce the influence of class imbalance on the decision boundaries, random oversampling was applied to the training data. Synthetic oversampling methods were not used because artificially interpolated feature vectors could distort the characteristics of the measured AE signals. Instead, duplication-based random oversampling was adopted to preserve the original feature distributions. For the tensile dataset, oversampling was performed separately within each training fold, and each class was increased to 47 training samples. Random oversampling was applied only to the tensile dataset; no class-balancing procedure was applied to the V-bending dataset.

The Random Forest model was constructed using decision trees as base learners. A total of 100 trees were used, and the maximum number of splits for each tree was set to 20. Five-fold cross-validation was performed to evaluate classification performance on the present dataset. The hyperparameters of the Random Forest classifier are summarized in Table 3.

### 2.6. Method for CWT Image-Based Clustering of Fracture-Associated AE Events

Fracture-associated AE signals generally occur much less frequently than plastic-deformation-related signals and exhibit irregular high-energy burst characteristics. In tensile tests, fracture occurs abruptly within a short time interval; therefore, such fracture-associated events account for only a small portion of the entire AE dataset. Owing to this sparsity and abruptness, entropy-based stage segmentation alone may not effectively isolate individual fracture-associated signals. Therefore, an additional CWT image-based clustering procedure was introduced in this study.

Unlike the deformation-stage classification, where sub-windowed statistical feature vectors were used, fracture signal clustering was performed at the individual AE-event level. Each AE event in the unstable deformation stage was converted into a CWT image, and the same 17 quantitative features described in Section 2.5 were extracted from each image. These features were used to represent the time–frequency characteristics of each AE event.

To examine whether fracture-associated AE events formed a distinguishable group within the unstable deformation stage, principal component analysis (PCA) was first applied to the extracted feature vectors for visualization. Subsequently, HDBSCAN was applied to the AE events for unsupervised clustering using MATLAB R2024a (The MathWorks, Inc., Natick, MA, USA). HDBSCAN was selected because it can identify clusters based on local data density without requiring the number of clusters to be specified in advance. This property is suitable for detecting sparse fracture-associated AE events, which may appear as a minority cluster within the overall AE dataset.

The HDBSCAN hyperparameters were set to min_cluster_size = 3, min_samples = 2, and cluster_selection_epsilon = 17.0, as summarized in Table 4. The HDBSCAN parameters were empirically selected based on preliminary clustering trials. Because only 49 AE events were analyzed near final fracture, the stability of the clustering results with respect to parameter variation was not fully established. The resulting clusters were interpreted by comparing representative CWT images and their signal characteristics.

## 3. AE Signals During Tensile and V-Bending Deformation

### 3.1. Tensile Deformation of 1 mm Thick Sheet

Understanding the fundamental characteristics of AE signals generated during material deformation is essential for subsequent signal analysis. For this purpose, AE signals obtained during tensile testing of the 1 mm thick AZ31B magnesium alloy sheet were examined.

**Figure 5:**
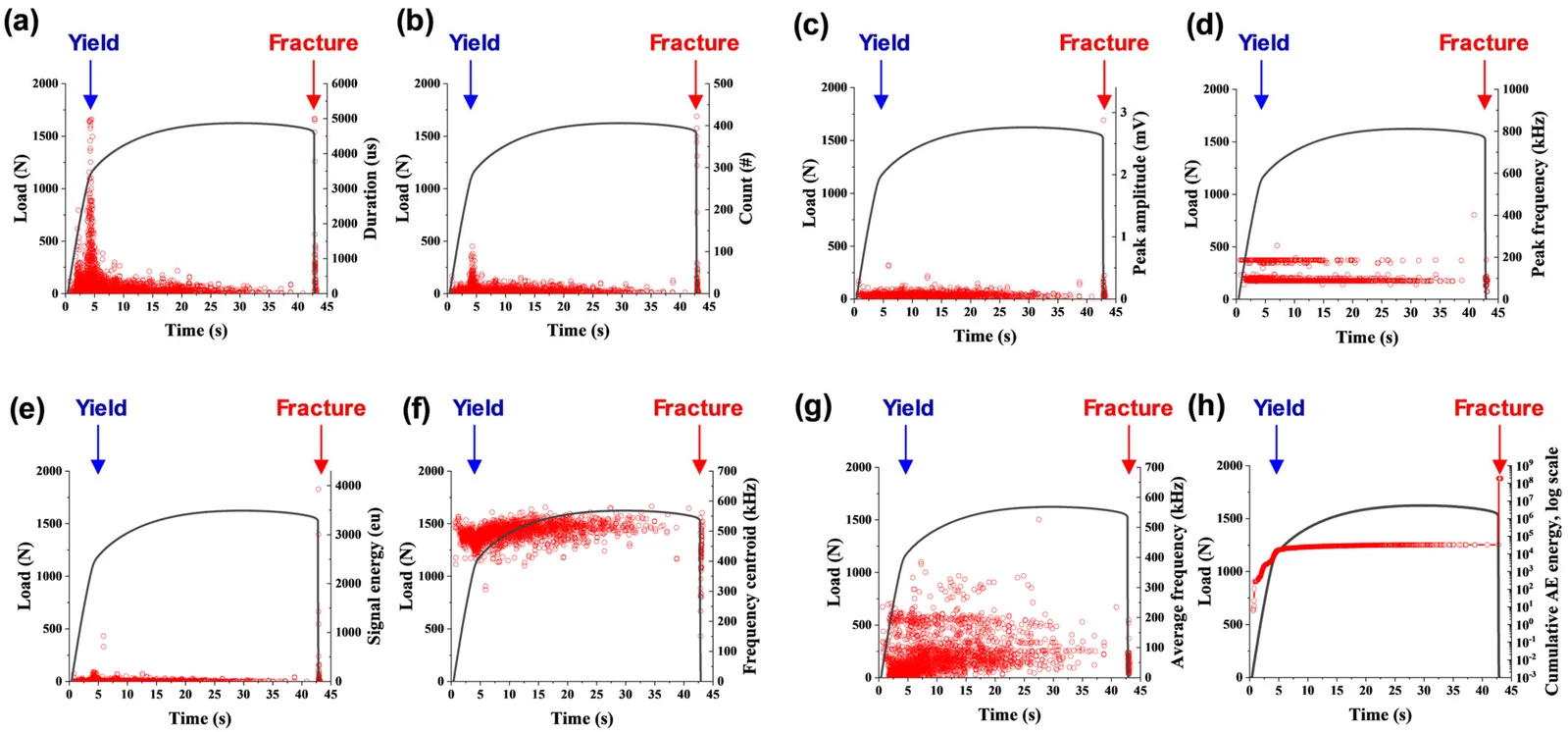
AE parameters measured during tensile deformation of the 1 mm thick AZ31B sheet: (**a**) duration, (**b**) count, (**c**) peak amplitude, (**d**) peak frequency, (**e**) signal energy, (**f**) frequency centroid, (**g**) average frequency, and (**h**) cumulative AE energy. The blue and red arrows indicate the yielding point and final fracture, respectively.

Figure 5 presents the major AE parameters measured during the tensile deformation process. During tensile deformation, the material undergoes elastic deformation, yielding, plastic deformation, necking, and final fracture. In AZ31B magnesium alloy, AE activity begins to increase near the transition from elastic to plastic deformation, and a similar behavior was also observed in our previous study [20]. In the yielding region, a large number of AE hits are generated intensively, resulting in a high count value. At the same time, the duration, peak amplitude, and signal energy also tend to increase. In contrast, the frequency centroid shows a decreasing tendency, while the cumulative energy increases rapidly due to the accumulation of AE events. After yielding, both the occurrence frequency and energy of AE signals generally decrease. Subsequently, as necking progresses, localized deformation becomes more severe, and high-energy AE signals are generated again near final fracture [20].

The AE signals generated during yielding and fracture exhibit distinct characteristics. In the yielding region, numerous AE hits are generated continuously over a relatively long period. This is attributed to the onset of plastic deformation throughout the gauge section of the specimen. In contrast, during fracture, AE signals with high amplitude and energy are concentrated within a very short period due to rapid crack initiation and propagation. Fracture-associated AE signals also exhibited longer durations and higher amplitudes, signal energies, and count values than those observed during yielding.

**Figure 6:**
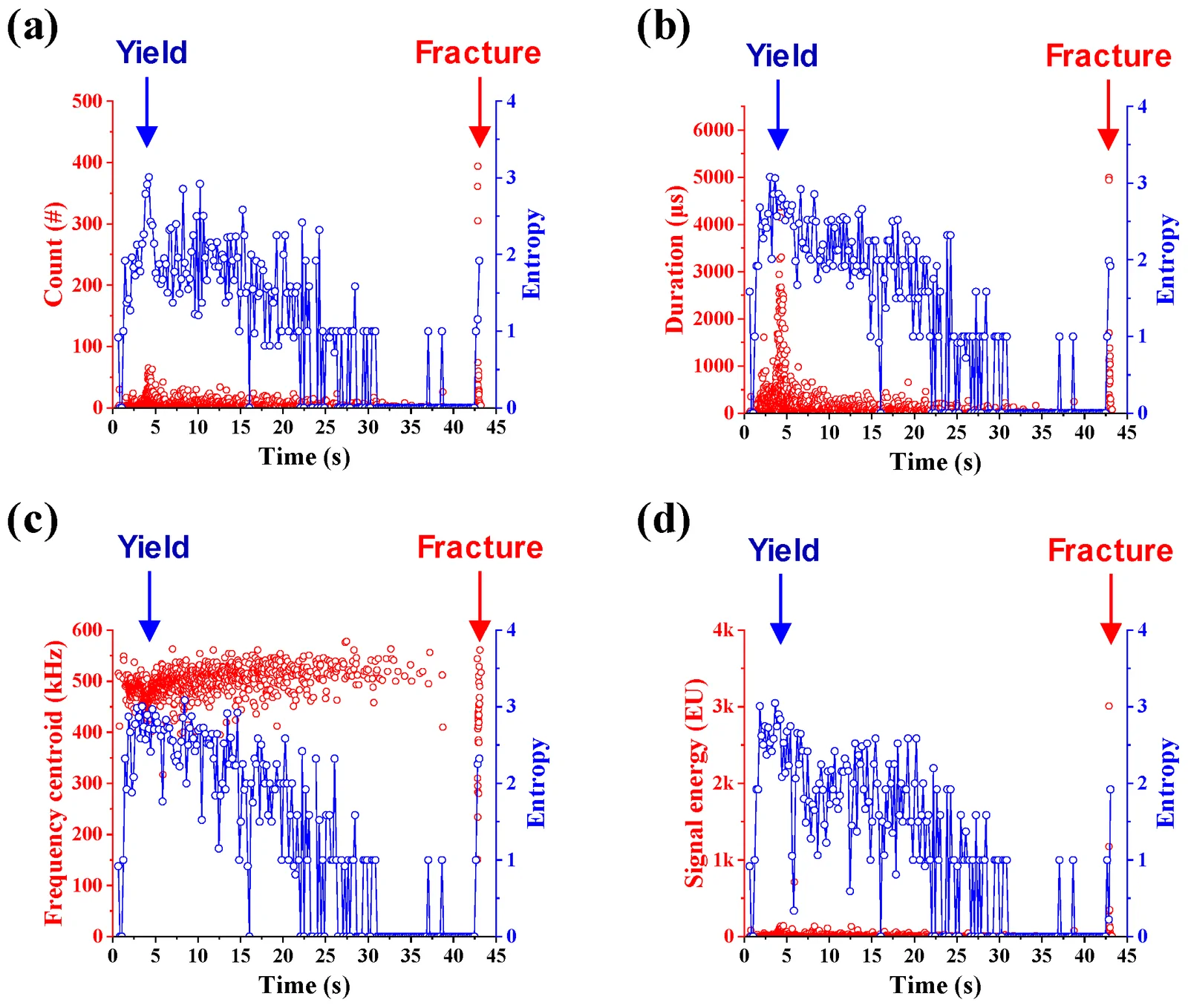
Shannon entropy histories of (**a**) count, (**b**) duration, (**c**) frequency centroid, and (**d**) signal energy during tensile deformation of the 1 mm thick AZ31B sheet. The blue and red arrows indicate the yielding point and final fracture, respectively.

Figure 6 shows the Shannon entropy calculated from AE signals generated during tensile deformation using a sliding window with a duration of 0.2 s. Shannon entropy exhibits high values in the regions where yielding begins and final fracture occurs. This behavior is attributed to the broad distribution of AE signal characteristics, such as amplitude, energy, and duration, during yielding and final fracture. In other words, AE hits with various magnitudes and signal features are generated simultaneously during yielding and fracture, leading to increased uncertainty and complexity in the signal distribution. In contrast, entropy decreases during the time interval of approximately 33–42 s, which can be attributed to the reduced occurrence frequency and lower variability of AE signals in this region.

**Figure 7:**
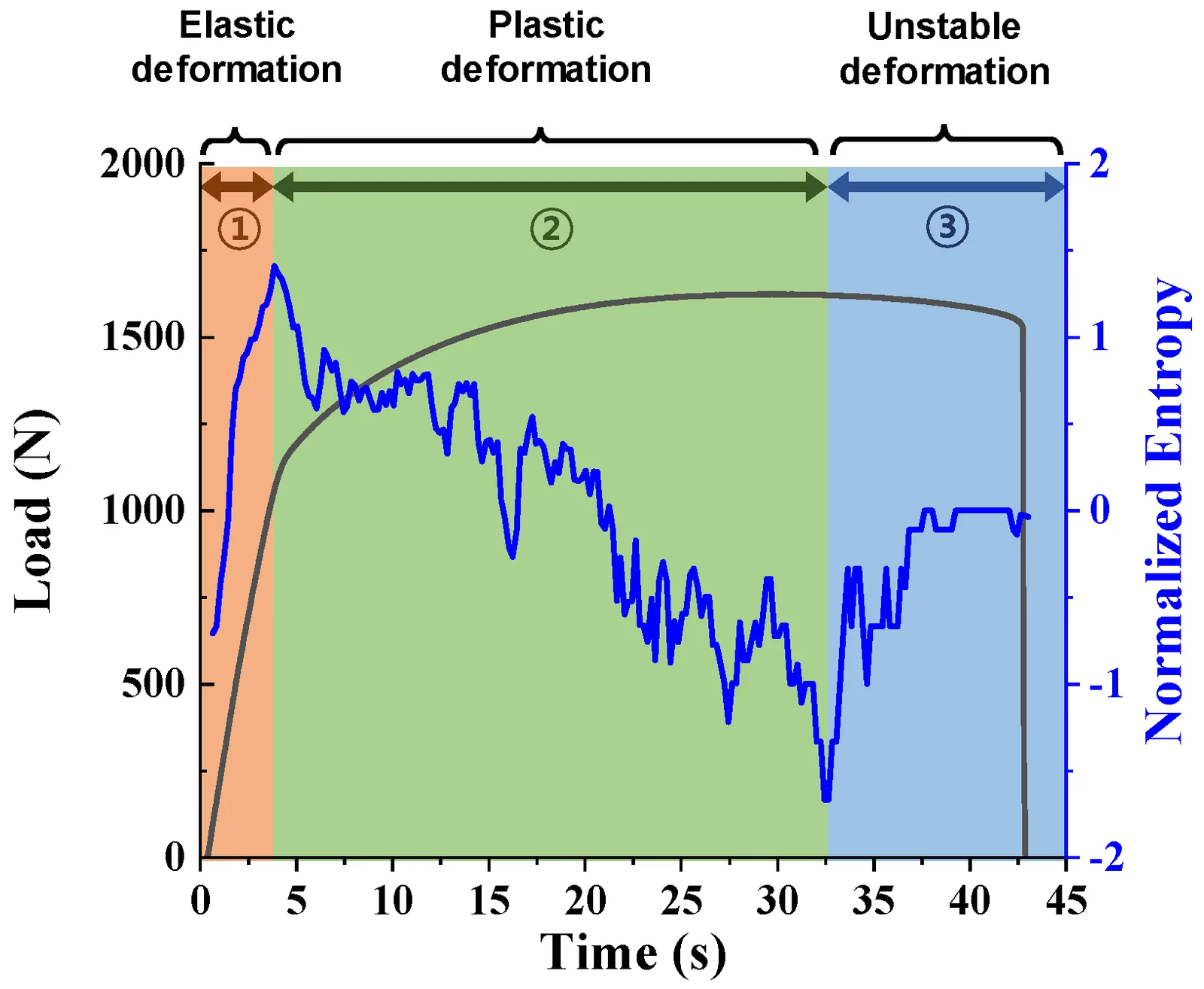
Segmentation of tensile deformation stages based on the normalized mean entropy curve.

The entropy values of the four AE parameters were normalized using z-score normalization. Figure 7 shows the segmentation of tensile deformation stages based on the normalized mean entropy curve. The first peak in Shannon entropy is associated with the transition from elastic to plastic deformation. In the initial stage, the Shannon entropy gradually increases in the elastic deformation stage. This increase can be attributed to the progressive development of plastic deformation and localized stress concentration as the tensile load increases, which results in a more complex distribution of AE signal characteristics. Therefore, the region before this peak was defined as the elastic deformation stage in this study.

After the first entropy peak, the Shannon entropy gradually decreases. This behavior indicates that, following yielding, plastic deformation continues mainly within regions that have already undergone plastic deformation, while the formation of newly yielded regions becomes less significant. Consequently, the AE response becomes more uniform, resulting in a reduction in signal complexity and entropy. Subsequently, the entropy starts to increase again at approximately 33 s. This increase is associated with the onset of deformation localization and necking. As strain becomes concentrated within a limited region and local damage begins to accumulate, AE signals with more diverse characteristics are generated. Therefore, this region was defined as the unstable deformation stage preceding final fracture.

### 3.2. V-Bending Deformation of 3 mm Thick Sheet

**Figure 8:**
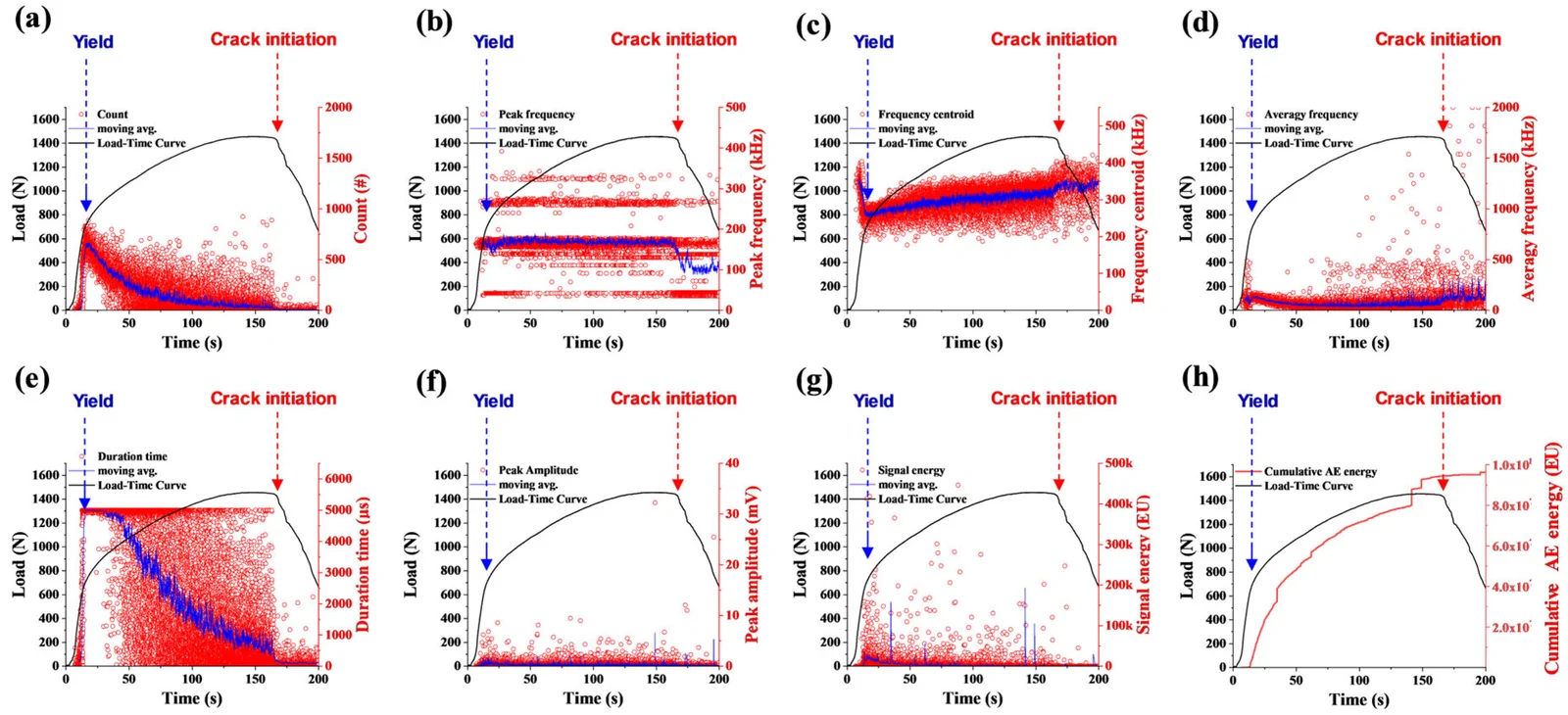
AE parameters measured during V-bending of the 3 mm thick AZ31B sheet: (**a**) duration, (**b**) count, (**c**) peak amplitude, (**d**) peak frequency, (**e**) signal energy, (**f**) frequency centroid, (**g**) average frequency, and (**h**) cumulative AE energy. The blue and red arrows indicate the yielding point and crack initiation, respectively.

Acoustic emission (AE) signals and Shannon entropy were analyzed during the V-bending deformation of a 3 mm thick AZ31B magnesium alloy sheet. Figure 8 presents the load–time curve and AE parameters during the bending deformation. During bending, compressive stress is induced on the inner surface, while tensile stress develops on the outer surface. Due to the relatively low ductility of AZ31B, fracture initiates in the outer tensile region near the center of the specimen, where deformation is highly concentrated.

As punch displacement increased during the bending process, the load gradually increased and reached a maximum value of approximately 1500 N at around 150 s. At this point, shallow cracks were observed on the outer surface of the specimen, and thus this point was identified as the crack initiation point. High strain and stress were concentrated in the outer tensile region, leading to increasingly localized deformation before crack initiation. Once the accumulated damage reached a critical level, microcracks initiated on the outer surface, and the region capable of accommodating deformation progressively became more localized [28]. From an AE perspective, frequent AE events were generated during this damage accumulation process, resulting in a rapid increase in the hit count. However, after crack initiation, deformation and damage became localized around the crack region, leading to a reduction in AE activity and a corresponding decrease in the hit count growth rate.

After approximately 170 s, the crack clearly propagated along the width direction of the specimen, resulting in a rapid decrease in load. As the test continued, the specimen could no longer accommodate bending deformation over the entire region, and residual deformation became concentrated only in a narrow region near the crack. Consequently, the load decreased with a steeper slope, and AE events were almost absent. At around 190 s, the load approached zero, indicating complete failure of the specimen.

Compared with tensile deformation, V-bending produced a more gradual AE response because plastic deformation developed locally near the bending center and outer tensile surface. AE activity was concentrated during plastic deformation and damage accumulation before crack initiation, and subsequently decreased as crack propagation localized deformation. In contrast, tensile fracture occurred abruptly and generated a rapid increase in AE activity immediately before final failure.

**Figure 9:**
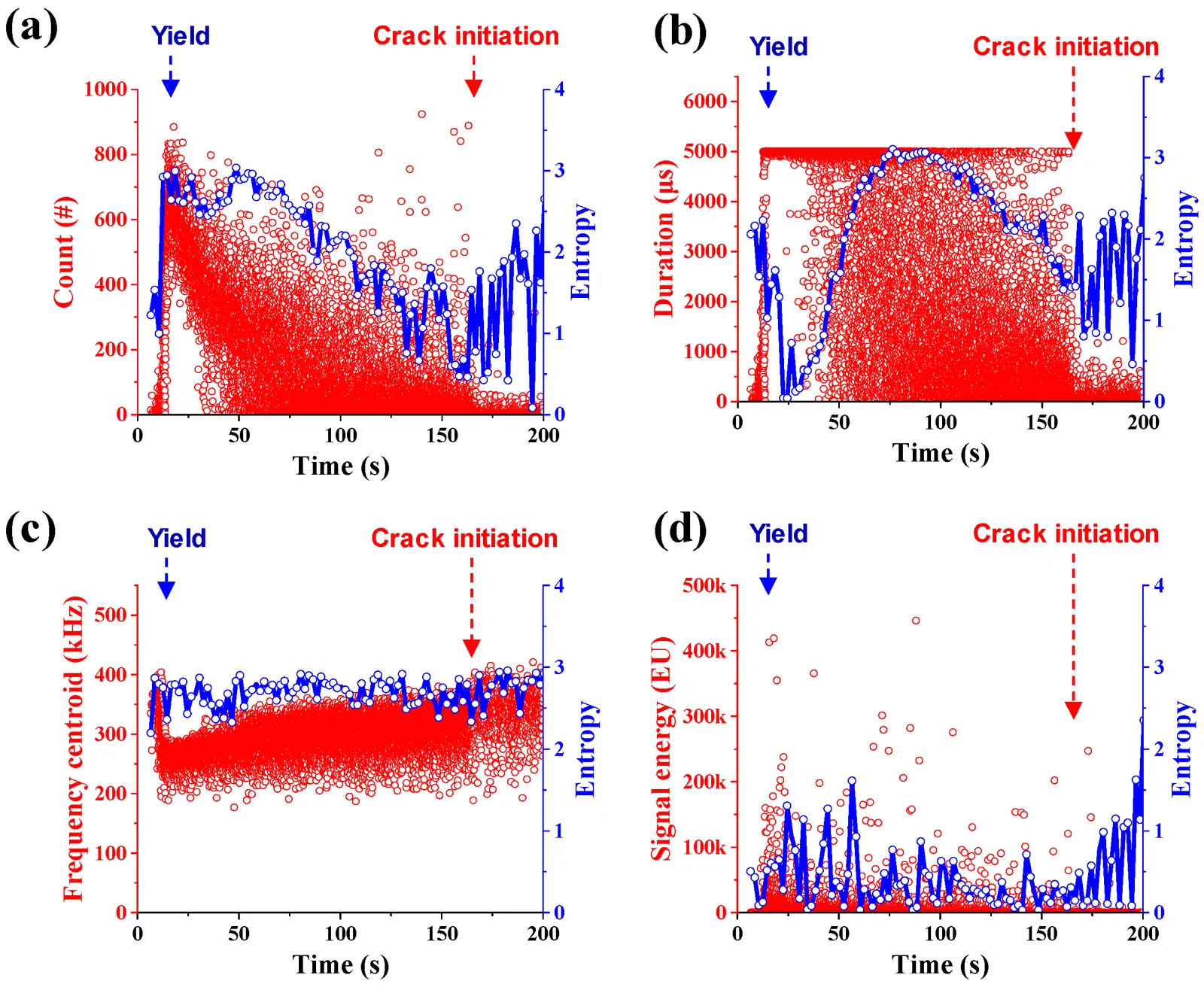
Shannon entropy of (**a**) count, (**b**) duration, (**c**) frequency centroid, and (**d**) signal energy during V-bending of the 3 mm thick AZ31B sheet. The blue and red arrows indicate the yielding point and crack initiation, respectively.

Shannon entropy analysis was performed on the AE events generated during the V-bending test using four representative AE parameters: count, duration, signal energy, and frequency centroid. The entropy of each parameter was calculated using a sliding window with a duration of 2 s, and the results are presented in Figure 9. The entropy values of the individual AE parameters exhibited different response patterns and served as meaningful indicators reflecting the temporal evolution of AE signals during the bending process.

The entropy of count increased rapidly during the early stage of deformation and remained at a relatively high level before gradually decreasing after approximately 75 s. After approximately 155 s, the entropy fluctuated again and showed a partial increase. However, this behavior is considered to be associated with irregular high-energy signals occurring in the later stage of the test rather than an increase in AE activity. The entropy of duration initially exhibited relatively high values, decreased gradually, and then increased again after approximately 22 s, reaching its maximum value near 75 s. Subsequently, it decreased rapidly and exhibited irregular fluctuations after approximately 155 s. The entropy of the frequency centroid remained relatively stable throughout the entire test, indicating that the temporal variation in the frequency distribution was limited during the bending process. In contrast, the entropy of signal energy exhibited irregular fluctuations throughout the test. In particular, intermittent high-energy AE events occurring after approximately 155 s resulted in an increase in the entropy calculated from signal energy.

The re-increase in entropy during crack initiation and propagation is attributed to the localization of deformation and the emergence of irregular AE responses associated with crack growth. While plastic deformation generates relatively repetitive AE characteristics, crack propagation introduces intermittent and non-uniform signal patterns, increasing the uncertainty of AE parameter distributions.

Since each AE parameter exhibited a different entropy trend, the complexity of the AE response could not be sufficiently represented by a single parameter. Therefore, following the same procedure used in the tensile test, the entropy values of the four parameters were normalized using z-score normalization and subsequently averaged to obtain a normalized mean entropy curve representing the overall evolution of AE signals.

**Figure 10:**
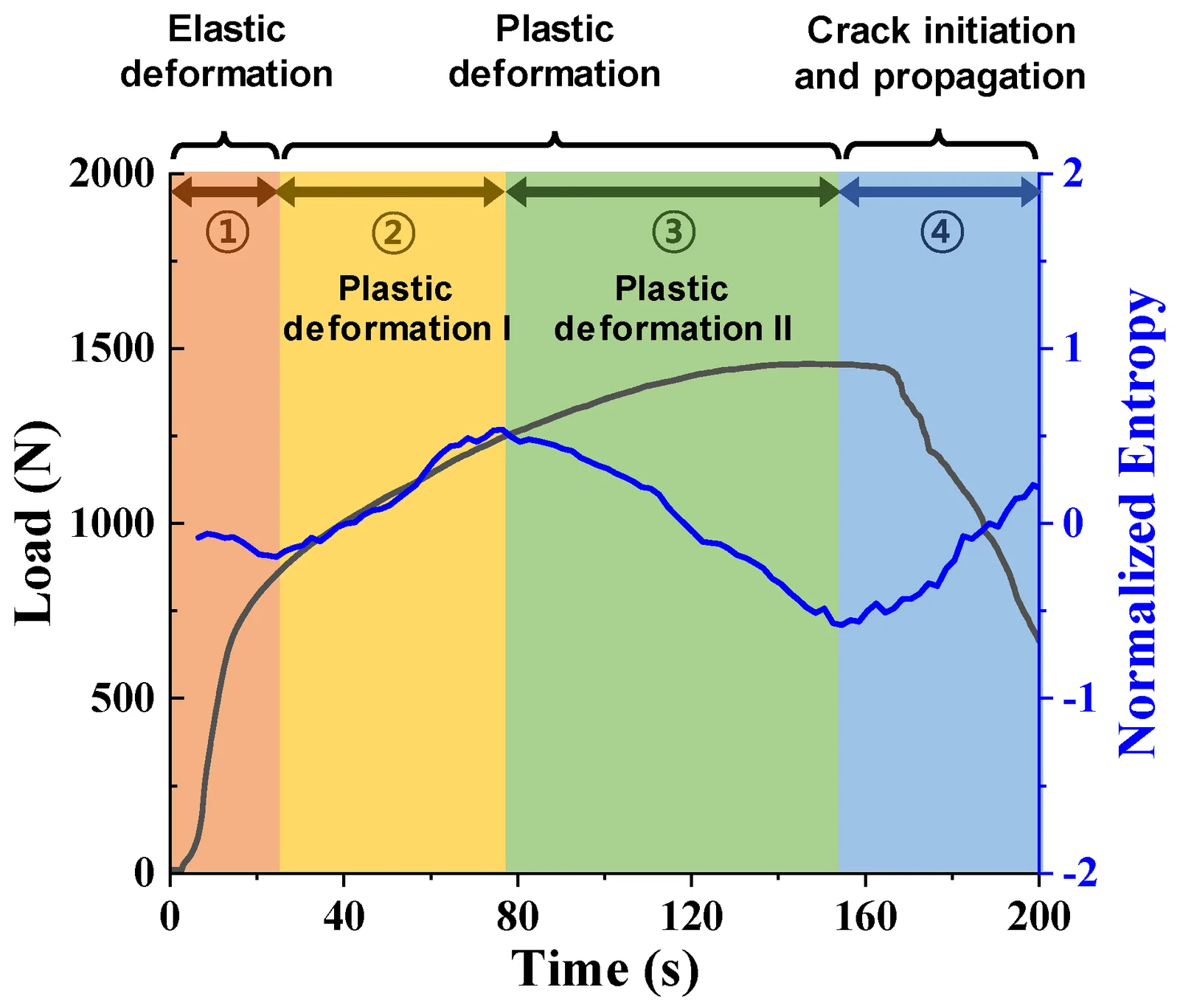
Segmentation of V-bending deformation stages based on the normalized mean entropy curve for the 3 mm thick sheet. Plastic deformation I and II represent entropy-defined sub-stages within the overall plastic deformation stage rather than distinct physical damage mechanisms.

Figure 10 shows the segmentation of the V-bending process of the 3 mm thick AZ31B sheet based on the normalized mean entropy curve. Four characteristic stages were identified from the entropy trend: elastic deformation, plastic deformation I, plastic deformation II, and crack initiation and propagation. Since plastic deformation I and II both correspond to the plastic deformation process, the overall bending deformation was ultimately categorized into three stages: elastic deformation, plastic deformation, and crack initiation and propagation.

During the elastic deformation stage, the load increased almost linearly, while only a limited number of AE events were detected. Accordingly, the normalized mean entropy remained at a low level, indicating a stable stress state before significant AE activity was initiated.

In the plastic deformation I stage, the region undergoing plastic deformation gradually expanded as bending deformation progressed. As AE events were generated from various locations and the distributions of AE parameters became more complex, the normalized mean entropy exhibited an increasing trend. In contrast, during the plastic deformation II stage, deformation became concentrated within previously deformed regions rather than spreading into new areas. Consequently, the region generating AE signals became progressively restricted, and the signal characteristics became more uniform, resulting in a decrease in entropy. Although the entropy trends differed between these two stages, both corresponded to plastic deformation and were therefore integrated into a single plastic deformation stage.

During the crack initiation and propagation stage, the load decreased after reaching its maximum value, and cracks initiated at the outer tensile surface and gradually propagated through the sheet thickness. In this stage, AE signals were no longer distributed throughout the plastic deformation stage but became concentrated near the crack tip and its surrounding area. As a result, AE events occurred intermittently and locally, exhibiting irregular signal characteristics associated with crack propagation. These changes increased the uncertainty of the AE parameter distributions, causing the normalized mean entropy to increase again. Therefore, the variation in normalized mean entropy effectively reflects the expansion and localization of plastic deformation, as well as the localization of AE activity associated with crack propagation.

## 4. Classification of the Deformation Characteristics

### 4.1. Random Forest Classification of Deformation Stages

The classification dataset was constructed by assigning entropy-defined stage labels to the sub-windowed CWT feature vectors. For the tensile test, the AE data were divided into three stages: elastic deformation, plastic deformation, and unstable deformation. Twenty consecutive AE events were grouped into one sub-window, and a 51-dimensional feature vector was generated from the extracted CWT image features. These labeled sub-window feature vectors were used as input data for the Random Forest classifier.

Random Forest classification was performed for the tensile deformation of the 1 mm specimen, for which consistent entropy-defined stage labels were obtained. A total of 1225 AE signals were obtained from the tensile deformation test of the 1 mm specimen, resulting in 61 sub-windows. The model performance was evaluated using 5-fold cross-validation. The dataset was divided into five subsets; in each iteration, four subsets were used for training, and the remaining subset was used for validation.

The classification performance was evaluated using precision, recall, and F1-score. Precision represents the proportion of correctly classified samples among all samples predicted as a specific class, whereas recall represents the proportion of correctly classified samples among all actual samples belonging to that class. The F1-score is the harmonic mean of precision and recall and provides a balanced measure of classification performance.

The classification metrics obtained from 5-fold cross-validation are summarized in Table 5. The average classification accuracy was 94.6%. In terms of class-wise performance, Class 1 showed a precision of 0.846, recall of 1.000, and F1-score of 0.916. Class 2 showed a precision of 1.000, recall of 0.958, and F1-score of 0.979. Class 3 was classified without error, with precision, recall, and F1-score values of 1.000. The macro-averaged precision, recall, and F1-score were 0.949, 0.986, and 0.965, respectively.

The V-bending deformation behavior of the 3 mm thick AZ31B sheet was analyzed using the same entropy-based framework. Entropy-based segmentation identified four characteristic stages. The deformation characteristics were categorized into three stages: elastic deformation (Class 1), plastic deformation (Class 2), and crack initiation/propagation (Class 3). The number of CWT images corresponding to AE events in Classes 1, 2, and 3 were 1642, 18,262, and 2871, respectively. These values were considerably larger than those obtained from the tensile test, indicating that bending deformation generated AE signals more continuously throughout the deformation process. In order to preserve temporal context and capture the overall evolution of AE responses, 20 consecutive CWT images were grouped into one window. Non-overlapping fixed-length windows were used, resulting in 81 windows for Class 1, 913 windows for Class 2, and 143 windows for Class 3. Random Forest classification was applied to assess the separability of the entropy-defined stages, and the 5-fold cross-validation results are summarized in Table 6.

The average classification accuracy obtained from the 5-fold cross-validation was 96.42%. Class 2 exhibited the highest classification performance, with a precision of 0.986, recall of 0.974, and F1-score of 0.980. Class 1 showed a recall of 0.951 and a precision of 0.918, indicating that most samples were correctly identified, although a limited number of sequences were misclassified into neighboring stages. Class 3 achieved an F1-score of 0.911, suggesting that the crack initiation and propagation stage could also be distinguished with reasonable consistency. The macro-averaged F1-score was 0.942.

These results indicate that CWT image-based Random Forest classification was effective in distinguishing the entropy-defined deformation stages for tensile deformation of the 1 mm thick AZ31B sheet and V-bending deformation of the 3 mm thick AZ31B sheet. However, the present classification results are limited to the deformation characteristics of the tested material and experimental conditions. Therefore, further validation using independent datasets with different thicknesses, materials, and forming conditions is required to assess the general applicability of the proposed approach.

### 4.2. CWT Image-Based Clustering Results for Fracture-Associated AE Events

Although entropy-based segmentation can identify the unstable deformation stage in the tensile test, fracture-associated AE events may still be mixed with non-fracture signals within this stage. Therefore, an additional CWT image-based clustering analysis was performed to examine whether fracture-associated AE signals could be separated from other AE signals.

This analysis was applied only to the tensile test, in which fracture occurred abruptly and generated high-energy AE signals within a short time interval. In contrast, fracture during V-bending progressed gradually as bending deformation continued, and distinct high-energy burst signals were not generated instantaneously. Therefore, the CWT image-based fracture signal clustering was limited to the tensile deformation case.

**Figure 11:**
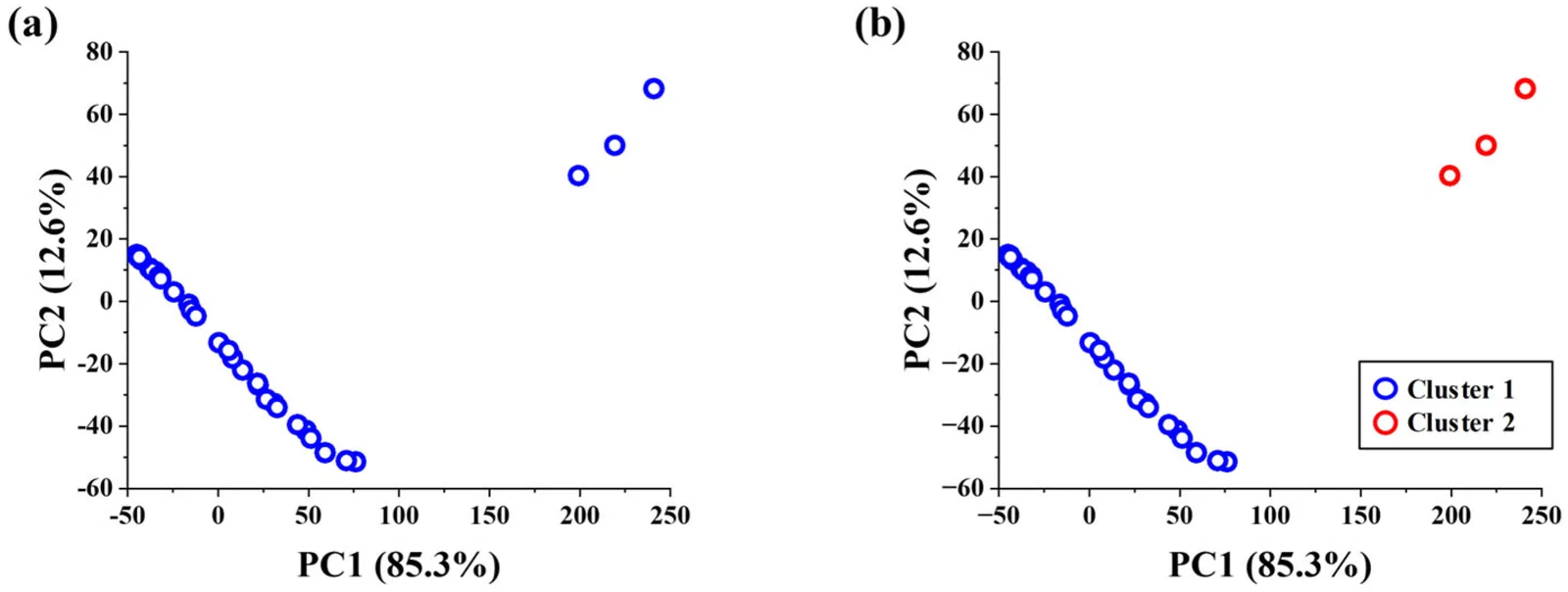
Distribution and clustering of AE events in the unstable deformation stage: (**a**) PCA projection in the PC1–PC2 space and (**b**) HDBSCAN clustering result.

A total of 49 AE events were identified in the tensile deformation of 1 mm thick AZ31B, corresponding to the necking and fracture stage during tensile deformation. Each AE event was converted into a time–frequency image using the same CWT procedure described in Section 2.4. To examine the internal distribution of AE events in this stage, 17 features were extracted from each CWT image, and principal component analysis (PCA) was performed. As shown in Figure 11a, partial separation was observed in the PC1–PC2 space, suggesting that AE events associated with ongoing plastic deformation and fracture occurred together during this stage.

Based on this feature distribution, HDBSCAN was applied to cluster the AE events in an unsupervised manner. The same hyperparameters defined in Section 2.6 were used: min_cluster_size = 3, min_samples = 2, and cluster_selection_epsilon = 17.0. As shown in Figure 11b, the AE events were separated into two major clusters, suggesting that the AE responses within Class 3 contained different groups exhibiting distinct characteristics in the extracted feature space.

**Figure 12:**
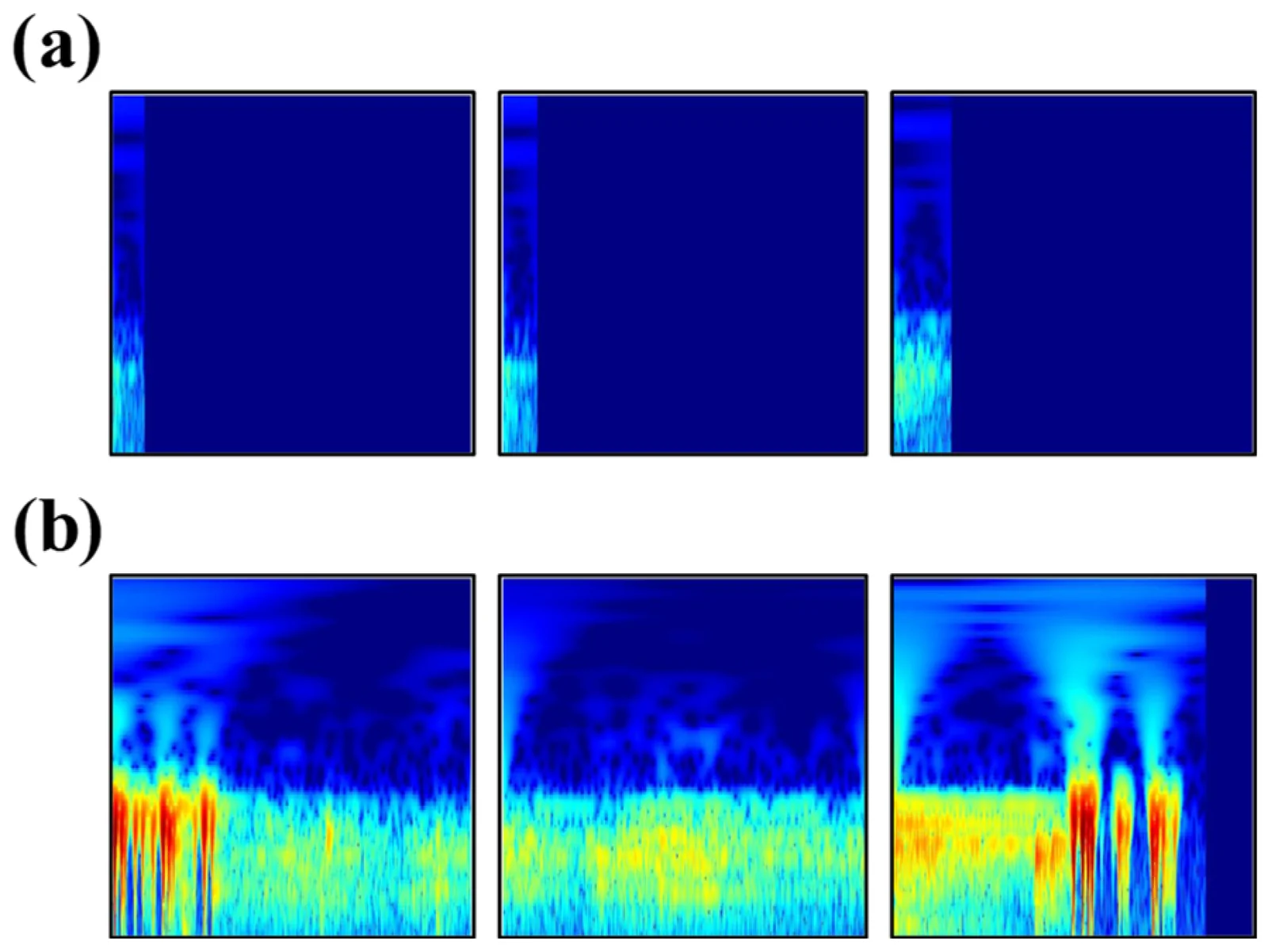
Representative CWT images corresponding to (**a**) low-intensity AE events and (**b**) high-intensity fracture-associated AE events.

Figure 12 presents representative CWT images of the AE events separated by HDBSCAN. The CWT images were generated using the same conditions described in Section 2.4, with a fixed time span of 5000 μs, a frequency range up to 600 kHz, and a logarithmic magnitude scale. Signals shorter than 5000 μs were zero-padded over the remaining time interval. The uniformly blue regions correspond to the zero-padded intervals where no AE waveform data were recorded. The CWT images in Cluster 1 exhibit relatively low signal-intensity AE responses, whereas Cluster 2 is characterized by higher signal intensity and longer duration. HDBSCAN separated a distinct group of high-energy AE events within the unstable deformation stage. Because these events occurred near final fracture and exhibited burst-type characteristics previously associated with crack initiation and fracture [20], they were interpreted as fracture-associated AE events. These results indicate that high-energy events occurring near final fracture formed a distinguishable cluster based on their CWT image features.

## 5. Discussion

The AE responses observed during tensile and V-bending deformation reflect differences in how plastic deformation develops under the two loading conditions. In the tensile test, plastic deformation spreads across the gauge region after yielding and then becomes localized as necking develops. AE activity decreases after yielding but becomes more variable as deformation localizes and fracture approaches. The corresponding entropy change therefore reflects the transition from distributed plastic deformation to localized deformation and final fracture. In V-bending, plastic deformation develops locally near the bending region and gradually expands as the punch displacement increases. Unlike tensile deformation, the continuous expansion of the plastically deformed region results in sustained AE activity and a different entropy trend. After crack initiation, the crack gradually propagates through the sheet thickness. The resulting intermittent AE responses increase the variability of the AE parameter distributions, leading to a renewed increase in entropy.

The present study focuses on signal-based characterization of deformation behavior rather than direct identification of microscopic deformation mechanisms. At room temperature, plastic deformation of AZ31B occurs through dislocation slip and deformation twinning, depending on the texture and loading direction. These mechanisms are likely to contribute to the AE activity observed during yielding and plastic deformation. However, the present AE data do not allow slip- and twinning-related signals to be separated. Therefore, the entropy change is interpreted as the overall AE response associated with plastic deformation, localization, and fracture, rather than with a specific deformation mechanism. The main advantage of the entropy-based approach is that it provides a single quantitative measure of the overall variation in multiple AE parameters. Because individual parameters have different scales and may change at different times, deformation-stage boundaries can be difficult to define consistently from each parameter alone. The normalized mean entropy combines these variations into a single index, providing a unified criterion for identifying changes between deformation stages.

Internal cross-validation showed that the entropy-defined stages in the 1 mm tensile and 3 mm V-bending datasets were distinguishable in the CWT-based feature space. This suggests that the sub-windowed CWT features captured differences in AE behavior among the identified deformation stages. However, because the classification labels were derived from entropy-based segmentation and no independent dataset was used for validation, these results should be regarded as evidence of within-dataset separability rather than as validation of a generalized predictive model. Similarly, the HDBSCAN cluster was interpreted as fracture-associated based on its temporal occurrence near final fracture and its AE characteristics, rather than on independent ground-truth identification. Therefore, the clustering result should be regarded as exploratory.

The detailed analysis focused on the 1 mm tensile and 3 mm V-bending specimens, which showed sufficient AE activity and relatively clear entropy trends. In the 1 mm bending test, very little AE activity was detected during most of the deformation, followed by a sudden increase near fracture, making entropy-based stage segmentation difficult. The 3 mm tensile specimen was also excluded from the detailed analysis because a distinct necking stage was not clearly observed before fracture. Because sheet thickness and specimen geometry can influence the mechanical response and AE activity, the selected tensile and V-bending cases were not interpreted as a direct quantitative comparison between different sheet thicknesses. Therefore, the selected cases should be considered representative examples rather than a complete validation across different thicknesses and deformation modes. As a next step, the proposed approach is currently being extended to a 2D draw-bending process, with further application to sheet metal stamping processes planned in future work.

This study was limited to quasi-static tensile and V-bending tests conducted under laboratory conditions. Measurement uncertainties related to the AE threshold, sensor coupling, and sensor position were not fully quantified and may have affected the measured AE parameters. In addition, because a resonant AE sensor was used, the frequency characteristics of the CWT images may have been influenced by the sensor response. In the representative CWT results, the dominant AE components were mainly concentrated within approximately 100–500 kHz, whereas components around 600 kHz and above exhibited relatively low magnitudes. Therefore, these high-frequency components are considered to have a limited contribution to the dominant measured time–frequency characteristics. Nevertheless, the CWT results were interpreted as characteristics of the recorded AE responses rather than as calibrated source spectra or direct signatures of specific microscopic deformation mechanisms. The present findings are limited to the investigated AZ31B specimens and loading conditions and should not be interpreted as validation of the proposed approach for other materials or forming conditions. Further studies using a calibrated wideband AE sensor, together with different sheet thicknesses, materials, loading rates, temperatures, and deformation conditions, are required to evaluate the high-frequency response and the broader applicability of the proposed approach.

## 6. Conclusions

This study proposed an entropy-based acoustic emission analysis framework for interpreting tensile and V-bending deformation behavior of AZ31B magnesium alloy sheets. Shannon entropy was calculated from multiple AE parameters to quantify temporal changes in AE signals, and the deformation processes were segmented based on the resulting entropy evolution.

For the 1 mm tensile deformation and 3 mm V-bending deformation cases, CWT image-based feature vectors were constructed by grouping consecutive AE events into sub-windows. Random Forest classification was used to evaluate the separability of the entropy-defined deformation stages in the extracted feature space. The results showed high internal 5-fold cross-validation accuracies of 94.6% and 96.42% for tensile and V-bending deformation, respectively, indicating that the entropy-defined stages had distinguishable CWT-based feature characteristics.

In addition, CWT image-based HDBSCAN was applied to the unstable deformation stage of the 1 mm tensile test. The clustering results identified a distinct group of high-intensity AE events near final fracture. Based on their temporal occurrence and previously reported AE characteristics, these events were interpreted as fracture-associated signals. This result suggests that CWT image-based clustering can supplement entropy-based stage segmentation by identifying fracture-associated AE responses within the unstable deformation stage.

The results demonstrate that Shannon entropy is useful for interpreting deformation-stage evolution in AZ31B magnesium alloy sheets, while CWT-based feature analysis provides additional information on stage separability and fracture-associated signal characteristics. However, the present results are limited to the tested material, thicknesses, and experimental conditions. Further validation using independent datasets with different thicknesses, materials, and forming conditions is required to assess the broader applicability of the proposed approach.

## Figures and Tables

**Table 1:** Acquisition settings of the AE sensor system.

Equipment	Topic	Values
AE sensor	Resonant frequency	150 kHz
Pre-amplifier	Input frequency range	1 kHz–1 MHz
	Gain	40 dB
	Power	28 Vdc
Digital acquisition system (DAQ)	Sampling rate	2 MS/s
	Threshold	40 dB
	Band-pass filter	20–1000 kHz
	Hit definition time (HDT)	400 μs
	Peak definition time (PDT)	200 μs
	Hit lockout time (HLT)	4000 μs
	Maximum hit duration time (MHD)	5000 μs

**Table 2:** Quantitative features extracted from CWT images for Random Forest classification.

Category	Feature Items
Statistical Features	Mean, Standard Deviation, Skewness, Kurtosis, Entropy, Maximum value
Texture Features (GLCM)	Contrast, Correlation, Energy, Homogeneity
Histogram-based Features	10th percentile, 25th percentile, 50th percentile (median), 75th percentile, 90th percentile
Frequency-domain Features	Mean of FFT spectrum amplitude, Maximum of FFT

**Table 3:** Hyperparameters of the Random Forest classifier.

Parameter	Value
Base learner	Decision tree
Number of trees	100 (NumLearningCycles = 100)
Maximum number of splits	20 (MaxNumSplits = 20)
Cross-validation scheme	5-fold

**Table 4:** Hyperparameters of the HDBSCAN algorithm.

Parameter	Value
Clustering algorithm	HDBSCAN
min_cluster_size	3
min_samples	2
cluster_selection_epsilon	17.0

**Table 5:** Internal 5-fold cross-validation performance of the Random Forest classifier for tensile deformation.

Class	Precision	Recall	F1-Score
Class 1 (Elastic deformation)	0.846	1.000	0.916
Class 2 (Plastic deformation)	1.000	0.958	0.979
Class 3 (Unstable deformation)	1.000	1.000	1.000
Macro average	0.949	0.986	0.965

**Table 6:** Internal 5-fold cross-validation performance of the Random Forest classifier for bending deformation.

Class	Precision	Recall	F1-Score
Class 1 (Elastic deformation)	0.918	0.951	0.934
Class 2 (Plastic deformation)	0.986	0.974	0.980
Class 3 (Crack initiation and propagation)	0.887	0.937	0.911
Macro average	0.930	0.954	0.942

## Data Availability

The original contributions presented in this study are included in the article. Further inquiries can be directed to the corresponding author.
